# Institute for Clinical and Economic Review's role in the US health care system: Centering the patient perspective

**DOI:** 10.1093/haschl/qxaf071

**Published:** 2025-04-08

**Authors:** Sarah K Emond, Catherine K Fischer

**Affiliations:** Institute for Clinical and Economic Review (ICER), 14 Beacon St, Suite 800, Boston, MA 02108, United States; Institute for Clinical and Economic Review (ICER), 14 Beacon St, Suite 800, Boston, MA 02108, United States

**Keywords:** Health technology assessment, independent, comparative effectiveness, value assessment, public process, prescription drug pricing, patient engagement, patient involvement, value-based pricing, patient access, stakeholder engagement, transparency, policy recommendations

## Abstract

Consequential health decisions in the United States are often made behind closed doors without input from patients and other critical stakeholders. The Institute for Clinical and Economic Review (ICER) brings drug pricing and coverage decisions into the open by conducting independent assessments of effectiveness and value that undergo a public process. While many stakeholders are invited to participate, ICER's dedicated patient engagement program demonstrates a strong commitment to those most impacted by the final report conclusions and recommendations. ICER believes that patients should have a voice in decisions that directly affect their ability to afford and access treatment.

## About ICER

Back in 2006, there were very few public conversations about how evidence can drive value in the U.S. health care system. Before that time, consequential health decisions were often made behind closed doors, inside health insurance companies and health care facilities, and absent the input of patients and other critical stakeholders. But ICER's founder, Dr. Steven D. Pearson, had a different idea: what if those important conversations happened in public and were driven by an independent assessment of effectiveness and value? Enter the Institute for Clinical and Economic Review (ICER).^1^

Even as ICER was experimenting with methods and finding its footing, its overarching mission was clear: to illuminate the tradeoffs the health care system was making due to limited resources, spark discussions about the outcomes that are most important to patients, and advance the idea that the benefits for patients of new technologies, like prescription drugs, should match the price being paid by society. It was clear to us then, as it is now, that a health care system that has prices aligned with value can deliver affordable access to patients, while driving the next wave of innovation.

Part of the work we do at ICER involves a multi-disciplinary science known as health technology assessment (HTA).^2^ The underpinnings of HTA ask seemingly simple questions: how do the added benefits of a new treatment (if any) compare with the existing standards of care, and how much are we, as society, paying for those benefits? To answer these questions, ICER undertakes a rigorous process, holding public-facing discussions with multiple stakeholders, such as patients and their families, clinical experts, the manufacturers of the therapies, and health plans and purchasers. We then conduct a robust systematic literature review to answer key questions about comparative clinical effectiveness, and translate those learnings into an economic model that is used to simulate long-term costs and outcomes and can help inform conversations around fair price. Our eight-month reviews offer multiple opportunities for public comment, and our research conclusions are subject to public deliberation and vote by our independent appraisal committees.^3^ This process is meant to produce actionable, independent research, freely available on our website, that can support the move to a more sustainable health care system.^1^

Our work is used by a wide range of public and private entities, including the Veteran's Health Administration, the Centers for Medicare and Medicaid Services, the Congressional Budget Office, state Medicaid departments, private health insurers, and the drug companies themselves.^4-7^ We are also a part of an international community of HTA practitioners and recently announced our Health Economics Method Advisory (HEMA) with Canada's Drug Agency (CDA-AMC) and England's National Institute for Health and Care Excellence.^8^ HEMA will critically and independently examine and assess new methods and processes to inform these and other HTA bodies about whether and how to implement them.

## The importance of patient engagement

As part of ICER's public process of producing comparative effectiveness reviews, ICER engages patients, clinicians, insurers, and manufacturers to ensure multistakeholder input into report development and public meeting deliberation.^9^ While all stakeholders are included from beginning to end of the drug review process, ICER's dedicated program for patient engagement has set a strong standard in the international HTA community, and demonstrates our particular commitment to those most impacted by the final report conclusions and recommendations.^10^ ICER believes that patients should have a seat at the table to influence decisions that directly affect their ability to afford and access treatment.

The patient community—comprising patients, caregivers, and patient advocacy organizations—is uniquely positioned to convey the many aspects of the lived experience with a certain disease or condition. As ICER reviews the existing clinical evidence for a new treatment option, it is often the details and context of the patient’s lived experience that are lacking from clinical trial data or conversations with other stakeholders. In assessing the comparative value of a new treatment, who better to include than those familiar with (and often taking) the existing treatments and having strong preferences for what a new treatment should offer? Only patients can share the daily impacts of the disease, the most burdensome symptoms, pros and cons of existing treatments, and the most meaningful outcomes for a new treatment to achieve. Patient voices are therefore critical to ICER's value assessment and critical to shaping how the US health system should think about drug development, comparative effectiveness, and pricing and coverage.

**Figure 1.**
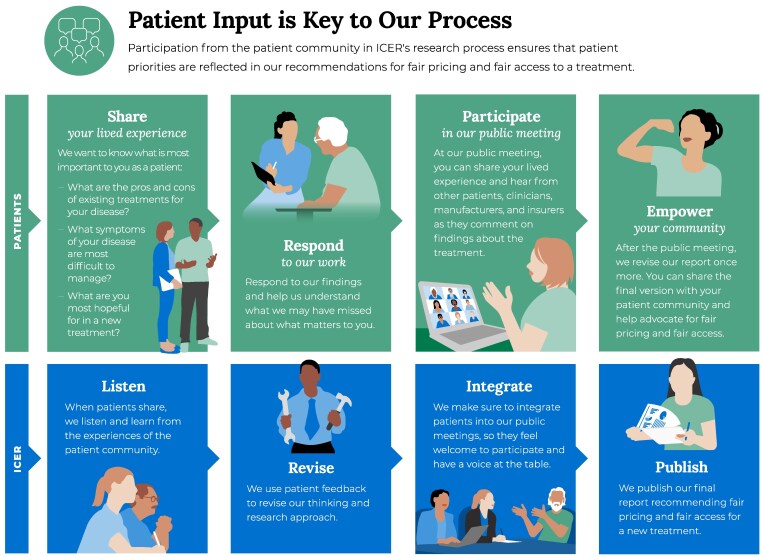
ICER’s patient engagement process.

ICER's patient engagement process is woven into the entirety of the drug review, with the patient community receiving an invitation to participate prior to public launch, followed by many opportunities for input and participation throughout. For those engaging with ICER for the first time, the process starts with an onboarding call to learn about ICER's role in the US health system and the purpose and impact of patient engagement in an ICER review. The process then continues through four phases during which the patient community is invited to (1) share their lived experience with ICER, (2) respond with feedback to ICER's work, (3) participate at ICER's public meeting, and (4) empower their community with evidence to advocate for fair pricing and access. Patient input is invited and accepted along all stages of the drug review process. Figure 1 below outlines ICER's patient engagement process and how ICER responds to patient input within each phase of the review.

ICER recognizes the often-limited time and resources for patients and patient advocacy groups to engage in the review process; therefore, opportunities for input are presented in different formats (eg, online Share Your Story Form, written comment letter, virtual meetings with ICER's team, small group patient or caregiver interview) to best accommodate each individual and group's capacity.^11^ We also recognize that, like any other stakeholder, the time that patients and caregivers take from other activities to spend with us is of great value, so we fairly compensate them for that time.

As ICER's patient engagement program has evolved over the recent years, a need for ongoing patient- and community-informed strategic advice has surfaced. This was met with the 2023 establishment of ICER's Patient Council, an external committee of patients representing a range of disease backgrounds, tasked with identifying areas for process improvement, and ensuring the diversity, inclusivity, and accessibility of ICER's outreach, process, and materials.^12^

Much like ICER's report development process, the patient engagement process follows an iterative and adaptive approach based on the needs and priorities of each patient community. ICER is committed to keeping the patient at the center of its mission and stakeholder engagement process, so that the final pricing and policy recommendations from the report are grounded in what matters most to patients about their disease and treatment options.

## About this series with *Health Affairs Scholar*

This new series with *Health Affairs Scholar* offers an opportunity to delve deeper into the policy implications of some of our recent research, with an emphasis on helping policymakers know how to make evidence-based policy choices. *Health Affairs Scholar's* focus on health care technology and population health overlaps with ICER's commitment to producing actionable research to address critical questions facing our health care system. This moment in health policy is fraught. Evidence and data may be under attack. We are grateful that *Health Affairs Scholar* is offering this venue for a continued conversation about how we bridge the research and practice gap.

Over the course of this series, you can expect to hear about the role of cost-effectiveness analysis in healthcare; balancing the tension between individual decision-making and population health; the patient and consumer perspective on de-escalating the arms race between higher prices and more access restrictions; and updates on the continued development and evolution of the methods to do this work.

This series is meant to serve as a place for conversation and action. No one knows what's in store for the U.S. health care system. But we know that centering conversations around evidence, benefits for patients, and cost-effectiveness will be one way we can navigate this uncertainty together.

## Supplementary Material

qxaf071_Supplementary_Data
