# Supplementary material for: Institute for Clinical and Economic Review's role in the US health care system: Centering the patient perspective
Source: Health Aff Sch. 2025 Apr 8;3(4):qxaf071. doi: 10.1093/haschl/qxaf071 (PMC12013816; doi:10.1093/haschl/qxaf071)
Supplement: qxaf071_Supplementary_Data [file qxaf071_supplementary_data.zip › ICMJE_COI Disclosure_Cat Koola Fischer.pdf]

ICMJE DISCLOSURE FORM

Date: 3/26/2025

Your Name: Catherine Koola Fischer

Manuscript Title: ICER's Role in the U.S. Health Care System: Centering the Patient Perspective

Manuscript Number (if known): Click or tap here to enter text.

In the interest of transparency, we ask you to disclose all relationships/activities/interests listed below that are related to the content of your manuscript. "Related" means any relation with for-profit or not-for-profit third parties whose interests may be affected by the content of the manuscript. Disclosure represents a commitment to transparency and does not necessarily indicate a bias. If you are in doubt about whether to list a relationship/activity/interest, it is preferable that you do so.

The author's relationships/activities/interests should be defined broadly. For example, if your manuscript pertains to the epidemiology of hypertension, you should declare all relationships with manufacturers of antihypertensive medication, even if that medication is not mentioned in the manuscript.

In item #1 below, report all support for the work reported in this manuscript without time limit. For all other items, the time frame for disclosure is the past 36 months.

		**Name all entities with whom you have this relationship or indicate none (add rows as needed)**	**Specifications/Comments (e.g., if payments were made to you or to your institution)**
**Time frame: Since the initial planning of the work**			
1	All support for the present manuscript (e.g., funding, provision of study materials, medical writing, article processing charges, etc.) **No time limit for this item.**	☐ None	
		Arnold Ventures	Grant awarded to ICER
		The Patrick and Catherine Weldon Donaghue Medical Research Foundation	Grant awarded to ICER
**Time frame: past 36 months**			
**2**	Grants or contracts from any entity (if not indicated in item #1 above).	☐ **None**	
		Blue Cross Blue Shield of MA	Grant awarded to ICER
		California Healthcare Foundation	Grant awarded to ICER
		Harvard Pilgrim Healthcare	Grant awarded to ICER
		Kaiser Foundation Health Plan Inc.	Grant awarded to ICER
		Massachusetts Health Policy Commission	Contract awarded to ICER
		Peterson Center on Healthcare	Grant awarded to ICER
		The Commonwealth Fund	Grant awarded to ICER
		The Peterson Health Technology Institute	Grant awarded to ICER
		US Department of Veteran Affairs	Contract awarded to ICER
		The Patrick and Catherine Weldon Donaghue Medical Research Foundation	Grant awarded to ICER

		Name all entities with whom you have this relationship or indicate none (add rows as needed)	Specifications/Comments (e.g., if payments were made to you or to your institution)
**3**	Royalties or licenses	☒ **None**	
**4**	Consulting fees	☒ **None**	
**5**	Payment or honoraria for lectures, presentations, speakers bureaus, manuscript writing or educational events	☒ **None**	
		Institute for Policy Advancement, Ltd.	Honoraria for speaking engagement; payment made to ICER
**6**	Payment for expert testimony	☒ **None**	
**7**	Support for attending meetings and/or travel	☐ **None**	
		Patient Engagement Open Forum / Synergist	Payment made to ICER
		Patients as Partners in Clinical Research	Payment made to ICER
**8**	Patents planned, issued or pending	☒ **None**	
**9**	Participation on a Data Safety Monitoring Board or Advisory Board	☒ **None**	
**10**	Leadership or fiduciary role in other board,	☒ **None**	

		Name all entities with whom you have this relationship or indicate none (add rows as needed)	Specifications/Comments (e.g., if payments were made to you or to your institution)
	society, committee or advocacy group, paid or unpaid		
**11**	Stock or stock options	☒ **None**	
**12**	Receipt of equipment, materials, drugs, medical writing, gifts or other services	☒ **None**	
**13**	Other financial or non-financial interests	☒ **None**	

Please place an "X" next to the following statement to indicate your agreement:

☒ I certify that I have answered every question and have not altered the wording of any of the questions on this form.
