# Supplementary material for: Institute for Clinical and Economic Review's role in the US health care system: Centering the patient perspective
Source: Health Aff Sch. 2025 Apr 8;3(4):qxaf071. doi: 10.1093/haschl/qxaf071 (PMC12013816; doi:10.1093/haschl/qxaf071)
Supplement: qxaf071_Supplementary_Data [file qxaf071_supplementary_data.zip › ICMJE_COI Disclosure_Sarah K. Emond.pdf]

ICMJE DISCLOSURE FORM

Date: 3/27/2025

Your Name: Sarah K. Emond

Manuscript Title: ICER's Role in the U.S. Health Care System: Centering the Patient Perspective

Manuscript Number (if known): Click or tap here to enter text.

In the interest of transparency, we ask you to disclose all relationships/activities/interests listed below that are related to the content of your manuscript. "Related" means any relation with for-profit or not-for-profit third parties whose interests may be affected by the content of the manuscript. Disclosure represents a commitment to transparency and does not necessarily indicate a bias. If you are in doubt about whether to list a relationship/activity/interest, it is preferable that you do so.

The author's relationships/activities/interests should be defined broadly. For example, if your manuscript pertains to the epidemiology of hypertension, you should declare all relationships with manufacturers of antihypertensive medication, even if that medication is not mentioned in the manuscript.

In item #1 below, report all support for the work reported in this manuscript without time limit. For all other items, the time frame for disclosure is the past 36 months.

		Name all entities with whom you have this relationship or indicate none (add rows as needed)	Specifications/Comments (e.g., if payments were made to you or to your institution)
		**Time frame: Since the initial planning of the work**	
1	All support for the present manuscript (e.g., funding, provision of study materials, medical writing, article processing charges, etc.) **No time limit for this item.**	☒ None	
		Click the tab key to add additional rows.	
		**Time frame: past 36 months**	
**2**	Grants or contracts from any entity (if not indicated in item #1 above).	☐ **None**	
		Arnold Ventures	Grant awarded to my institution
		Blue Cross Blue Shield of MA	Grant awarded to my institution
		California Healthcare Foundation	Grant awarded to my institution
		Harvard Pilgrim Healthcare	Grant awarded to my institution
		Kaiser Foundation Health Plan Inc.	Grant awarded to my institution
		Massachusetts Health Policy Commission	Contract awarded to my institution
		The Patrick and Catherine Weldon Donaghue Medical Research Foundation	Grant awarded to my institution
			Grant awarded to my institution
		The Commonwealth Fund	Grant awarded to my institution
		The Peterson Health Technology Institute/	Grant awarded to my institution
		US Department of Veteran Affairs	Contract awarded to my institution

		Name all entities with whom you have this relationship or indicate none (add rows as needed)	Specifications/Comments (e.g., if payments were made to you or to your institution)
**3**	Royalties or licenses	☒ **None**	
**4**	Consulting fees	☒ **None**	
**5**	Payment or honoraria for lectures, presentations, speakers bureaus, manuscript writing or educational events	☐ **None**	
		BMO Capital Markets	Honoraria paid to my institution for speaking engagement.
		American College of Rheumatology	Honoraria paid to my institution for speaking engagement.
		Magellan Health	Honoraria paid to my institution for speaking engagement.
		UC Irvine	Honoraria paid to my institution for speaking engagement.
**6**	Payment for expert testimony	☒ **None**	
**7**	Support for attending meetings and/or travel	☒ **None**	
		The Alliance of Community Health Plans (ACHP)	Expense reimbursement for travel to speak at a meeting.
		AHIP	Expense reimbursement for travel to speak at a meeting.
		Academy of Managed Care Pharmacy (AMCP)	Expense reimbursement for travel to speak at a meeting.
		Alliance for Regenerative Medicine (ARM)	Expense reimbursement for travel to speak at a meeting.
		American Society of Gene & Cell Therapy (ASGCT)	Expense reimbursement for travel to speak at a meeting.
		International Federation of Health Plans (iFHP)	Expense reimbursement for travel to speak at a meeting.
		International Society for Pharmacoeconomics and Outcomes Research (ISPOR)	Expense reimbursement for travel to speak at a meeting.
		Magellan	Expense reimbursement for travel to speak at a meeting.
		Multi-Regional Clinical Trials Center (MRCT)	Expense reimbursement for travel to speak at a meeting.
		National Academy of Medicine (NAM)	Expense reimbursement for travel to speak at a meeting.

		Name all entities with whom you have this relationship or indicate none (add rows as needed)	Specifications/Comments (e.g., if payments were made to you or to your institution)
		National Alliance of Healthcare Purchaser Coalitions	Expense reimbursement for travel to speak at a meeting.
		Oliver Wyman	Expense reimbursement for travel to speak at a meeting.
		PCMA	Expense reimbursement for travel to speak at a meeting.
		Sanofi	Expense reimbursement for travel to speak at a meeting.
		University of North Carolina (UNC)	Expense reimbursement for travel to speak at a meeting.
		UC Irvine	Expense reimbursement for travel to speak at a meeting.
		International Myeloma Foundation (IMF)	Expense reimbursement for travel to speak at a meeting.
**8**	Patents planned, issued or pending	☒ **None**	
**9**	Participation on a Data Safety Monitoring Board or Advisory Board	☒ **None**	
**10**	Leadership or fiduciary role in other board, society, committee or advocacy group, paid or unpaid	☒ **None**	
**11**	Stock or stock options	☒ **None**	
**12**	Receipt of equipment, materials, drugs, medical writing, gifts or other services	☒ **None**	
**13**	Other financial or non-financial interests	☐ None	
		Abbott	Dues paid to my institution for an annual policy summit put on by my institution.
		AHIP	Dues paid to my institution for an annual policy summit put on by my institution.

	Name all entities with whom you have this relationship or indicate none (add rows as needed)	Specifications/Comments (e.g., if payments were made to you or to your institution)
	Alnylam	Dues paid to my institution for an annual policy summit put on by my institution.
	Astra Zeneca	Dues paid to my institution for an annual policy summit put on by my institution.
	Bayer Healthcare	Dues paid to my institution for an annual policy summit put on by my institution.
	Blue Shield of CA	Dues paid to my institution for an annual policy summit put on by my institution.
	Boehringer-Ingelheim	Dues paid to my institution for an annual policy summit put on by my institution.
	Centene Pharmacy Solutions	Dues paid to my institution for an annual policy summit put on by my institution.
	Chiesi USA, Inc.	Dues paid to my institution for an annual policy summit put on by my institution.
	CRISPR Therapeutics	Dues paid to my institution for an annual policy summit put on by my institution.
	CVS	Dues paid to my institution for an annual policy summit put on by my institution.
	Eisai	Dues paid to my institution for an annual policy summit put on by my institution.
	Elevance Health	Dues paid to my institution for an annual policy summit put on by my institution.
	EQRx	Dues paid to my institution for an annual policy summit put on by my institution.
	Express Scripts	Dues paid to my institution for an annual policy summit put on by my institution.
	Genentech/Roche	Dues paid to my institution for an annual policy summit put on by my institution.
	GSK	Dues paid to my institution for an annual policy summit put on by my institution.
	Health Care Service Corporation	Dues paid to my institution for an annual policy summit put on by my institution.
	Humana	Dues paid to my institution for an annual policy summit put on by my institution.
	Kaiser Permanente	Dues paid to my institution for an annual policy summit put on by my institution.
	Karuna Therapeutics	Dues paid to my institution for an annual policy summit put on by my institution.
	LEO Pharma	Dues paid to my institution for an annual policy summit put on by my institution.
	Mallinckrodt Pharmaceuticals	Dues paid to my institution for an annual policy summit put on by my institution.
	Merck	Dues paid to my institution for an annual policy summit put on by my institution.
	National Pharmaceutical Council	Dues paid to my institution for an annual policy summit put on by my institution.
	Novartis	Dues paid to my institution for an annual policy summit put on by my institution.
	Novo Nordisk	Dues paid to my institution for an annual policy summit put on by my institution.
	Otsuka Pharmaceuticals	Dues paid to my institution for an annual policy summit put on by my institution.

		Name all entities with whom you have this relationship or indicate none (add rows as needed)	Specifications/Comments (e.g., if payments were made to you or to your institution)
		Point32Health	Dues paid to my institution for an annual policy summit put on by my institution.
		Premera Blue Cross	Dues paid to my institution for an annual policy summit put on by my institution.
		Prime Therapeutics	Dues paid to my institution for an annual policy summit put on by my institution.
		Regeneron	Dues paid to my institution for an annual policy summit put on by my institution.
		Sanofi	Dues paid to my institution for an annual policy summit put on by my institution.
		Sun Life Financial	Dues paid to my institution for an annual policy summit put on by my institution.
		United Healthcare	Dues paid to my institution for an annual policy summit put on by my institution.

Please place an "X" next to the following statement to indicate your agreement:

☒ I certify that I have answered every question and have not altered the wording of any of the questions on this form.
